# The Intersections of Ethnicity, Nativity Status and Socioeconomic Position in Relation to Periodontal Status: A Cross-Sectional Study in London, England

**DOI:** 10.3390/ijerph181910519

**Published:** 2021-10-07

**Authors:** Syeda Ammara Shaharyar, Eduardo Bernabé, Elsa Karina Delgado-Angulo

**Affiliations:** 1 Dental Public Health Group, Faculty of Dentistry, Oral & Craniofacial Sciences, King’s College London, London SE5 9RS, UK; syeda.shaharyar@kcl.ac.uk (S.A.S.); elsa.delgado_angulo@kcl.ac.uk (E.K.D.-A.); 2 Departamento Académico de Odontología Social, Universidad Peruana Cayetano Heredia, Lima 15102, Peru

**Keywords:** ethnic groups, migration status, socioeconomic position, periodontal disease

## Abstract

The role of migration as a social determinant of periodontitis has been overlooked. Intersectionality theory could help understand how immigration status interacts with other social determinants of health to engender inequalities in periodontitis. The objective of the present study was to evaluate whether ethnicity, nativity status and socioeconomic position intersect to structure social inequalities in periodontal status. Data from 1936 adults in a deprived and multi-ethnic area of London were analysed. The numbers of teeth with probing depth and clinical attachment loss were determined from clinical examinations. A matrix with 51 intersectional strata, defined according to ethnicity, nativity status and education, was created. A cross-classified multilevel analysis, with participants clustered within intersectional social strata, was performed to assess the extent to which individual differences in periodontal measures were at the intersectional strata level. A complex pattern of social inequalities in periodontal status was found, which was characterised by high heterogeneity between strata and outcome-specificity. The variance partition coefficient of the simple intersectional model, which conflated additive and interaction effects, indicated that 3–5% of the observed variation in periodontal measures was due to between-stratum differences. Moreover, the percentual change in variance from the simple intersectional to the intersectional interaction model indicated that 73–74% of the stratum-level variance in periodontal measures was attributed to the additive effects of ethnicity, nativity status and education. This study found modest evidence of intersectionality among ethnicity, nativity status and education in relation to periodontal status.

## 1. Introduction

Periodontitis is a plaque-induced, inflammatory disease that progressively affects the tissues supporting the teeth [1]. It is a common disease, affecting 10% of people globally [2]. As the disease progresses, it can affect function and aesthetics and lead to tooth loss [3]. The presentation of periodontitis is shaped by social circumstances, with greater prevalence and severity of disease among worse-off individuals [4,5]. There is also evidence of ethnic inequalities in periodontal disease, although not all ethnic minorities are at a disadvantage compared to the White population [6,7,8,9,10]. Ethnic inequalities are not accounted for by demographic characteristics, socioeconomic position (SEP) or behaviours [7,10].

The role of migration as a social determinant of health is often overlooked. Most immigrants face challenges upon arrival to a new country, such as language barriers and issues accessing housing, employment opportunities and health care, all of which can impact on health [11,12]. The acculturation framework has traditionally informed studies on migrant health, whereby the initial health advantage of immigrants after arrival to a new country dilutes as they transition to the lifestyles of the host population [13,14]. The acculturation framework ignores the role of structural forces, such as the social determinants of health and institutional racism, that affect the social and economic integration of migrants into the host society [15,16]. On the contrary, an intersectionality framework posits that multiple social dimensions/identities (such as ethnicity, immigration status and SEP) intersect at the individual level of day-to-day experiences to reflect the various interlocking systems of power, privilege and oppression at the structural level that perpetuate health inequalities (such as racism, xenophobia and classism) [17,18]. Recognising these multiple intersecting dimensions is paramount to understand how immigration status interacts with other social determinants of health to engender health inequalities [19,20,21].

A few studies have explored the interplay among ethnicity, immigration status and periodontitis [22,23]. The National Health and Nutrition Examination Survey (NHANES) 2011–2012 data showed that immigration status explained part of the ethnic inequalities in periodontitis among immigrants aged 30+ years. However, no differences in the prevalence of periodontitis between US-born, naturalised and non-citizens were noted [24]. Finally, NHANES 2013–2014 data showed that, among immigrants aged 30+ years, every ethnic minority was more likely than the White group to have periodontitis [25]. Little is known about the oral health of adult immigrants in other developed countries, including the United Kingdom (UK). Two earlier studies showed that native-born adults have more caries experience [26] and greater odds of having tooth loss but not toothache [27] compared to their foreign-born counterparts, with large variations by ethnicity. The objective of the present study was to evaluate whether ethnicity, nativity status and SEP intersect to structure social inequalities in periodontal conditions.

## 2. Materials and Methods

### 2.1. Study Population

This study used cross-sectional data from the East London Oral Health Inequalities (ELOHI) study, a mixed-methods project exploring the connections among area deprivation, ethnicity and oral health of families in Outer North East London (ONEL, which includes the boroughs of Barking and Dagenham, Redbridge and Waltham Forest). The area is largely deprived (the 3 boroughs are in the most deprived decile in England) and hosts a multi-ethnic population (37% ethnic minorities) of over 600,000 inhabitants [28].

Phase 1 of ELOHI was a population-based cross-sectional survey of adults, 16 to 65 years old, carried out in 2009–2010. A sample representative of the general non-institutionalised population in ONEL was recruited using stratified multistage random sampling. A record of all addresses in the area, stratified according to the number of wards in each borough, formed the sampling frame. At least 55 addresses were randomly chosen from every ward, to yield 3193 addresses. All addresses received postal invitations. Visits to nonrespondent households were organised to determine the nature of the premise and the age of household members. During this process, 457 commercial/vacant premises and 208 households with no adults in the target age band were excluded. The final sampling frame contained 2528 valid addresses, of which 1437 agreed to participate (57% response rate). In each selected household, a maximum of two adults were invited and all agreed to participate, yielding a sample of 2343 adults.

### 2.2. Assessment of Variables

The outcome measure, periodontal status, was indicated by the numbers of teeth with probing depth ≥4 mm and with clinical attachment loss ≥4 mm (henceforth referred to simply as PD and CAL, respectively). Clinical examinations were conducted at home, with participants seated on chairs, under artificial light from Daray light lamps, and using mirrors and CPI type C periodontal probes. All teeth, including third molars, were clinically examined. PD and CAL were measured at the mesial and distal sites of each tooth (buccally for upper teeth and lingually for lower teeth). One hundred and thirty-three participants received a duplicate examination by a senior examiner, within a two-week interval, to evaluate inter-examiner reliability. The Kappa score for PD was 0.57 and for CAL was 0.58 by tooth.

Intersectional strata were based on combinations of ethnicity, nativity status and SEP. Ethnicity was self-assigned from a list containing exhaustive and mutually exclusive ethnic categories. Participants from 9 ethnic groups were selected for this analysis as they are the main ethnicities in the country according to contemporaneous census data. They were Asian Indian, Pakistani, Bangladeshi and Other, Black African, Caribbean and Other, and White British and Other. Nativity status was determined from country of birth (native-born or foreign-born). SEP was indicated by the highest qualification achieved and grouped as follows: low (none and secondary school), medium (A-levels and/or technical qualifications) and high education (first and higher degrees). Demographic factors (sex and age) were also included in the analysis as potential confounders.

### 2.3. Data Modelling

We carried out a cross-classified multilevel analysis of individual heterogeneity and discriminatory accuracy (MAIHDA) [29,30], with participants (level 1) nested within intersectional social strata (level 2). To that end, a matrix of 51 intersectional social strata was created based on participants’ ethnicity, nativity status and education (9 × 2 × 3 = 54 minus 3 because foreign-born White adults were not included), and each stratum was assigned a unique identifier. In line with intersectionality theory, social dimensions in MAIHDA are treated as contextual factors and equally relevant to periodontal status [30]. Two multilevel negative binomial regression models were fitted for each periodontal measure, namely, the simple intersectional model and the intersectional interaction model [29]. The *simple intersectional model* is a null model with random intercepts for social strata. The between-strata variance in this model captures the total amount of variability in the periodontal measure between social strata. The model was also used to estimate the predicted numbers of teeth with PD and CAL for every intersectional social stratum. The *intersectional interaction model* includes the main effects of ethnicity, nativity status and education (and those of the confounders) as fixed effects to remove the additive effects of social strata. Thus, the stratum-level residuals are a measure of excess risk due to interaction, reflecting any two-way or higher interactions between them (the intersectional effect). The between-stratum variance in this model captures what remains of the variability in the periodontal measure after the additive effects of social strata and confounders are adjusted for. From each model, we estimated the variance partition coefficient (VPC) and the proportional change in variance (PCV). The VPC, which was estimated using formulas for negative binomial regression models [31], measures the percent of the total sample variance attributed to between-stratum variance. The PCV measures the percent of the between-stratum variance in the null model explained by the additive effects of social strata and confounders [29,32].

All multilevel models were built in MLwiN 3.05 [33], which was called from Stata 16 via the *runmlwin* command [34]. Estimations were conducted using Markov chain Monte Carlo (MCMC) methods [35], with quasi-likelihood methods used to provide starting values. We specified diffuse prior distributions for all parameters, 50,000 iterations, a burn-in period of 5000 and a thinning interval of 10 iterations. For each parameter, point estimates were the means of the MCMC chains and the 95% credible intervals (95% CI) were the 2.5th and 97.5th percentiles of the respective MCMC chains [32].

## 3. Results

Data from 2199 adults, across nine ethnic groups, were available for analysis. Of them, 236 were excluded for missing information on nativity status and education (n = 258 and 94, respectively). No differences between participants in the study sample and those excluded for missing data were noted. All 51 intersectional social strata were represented in the study sample, with 36 (71%) containing 10 or more participants and 30 (59%) containing 20 or more participants. On average, participants had 11.4 (SD: 9.9, range: 0–32) and 4.2 (SD: 6.6, range: 0–32) teeth with PD and CAL, respectively. The number of teeth with PD differed by ethnicity and nativity status, and the number of teeth with CAL differed by nativity status and education (Table 1).

**Figure 1:**
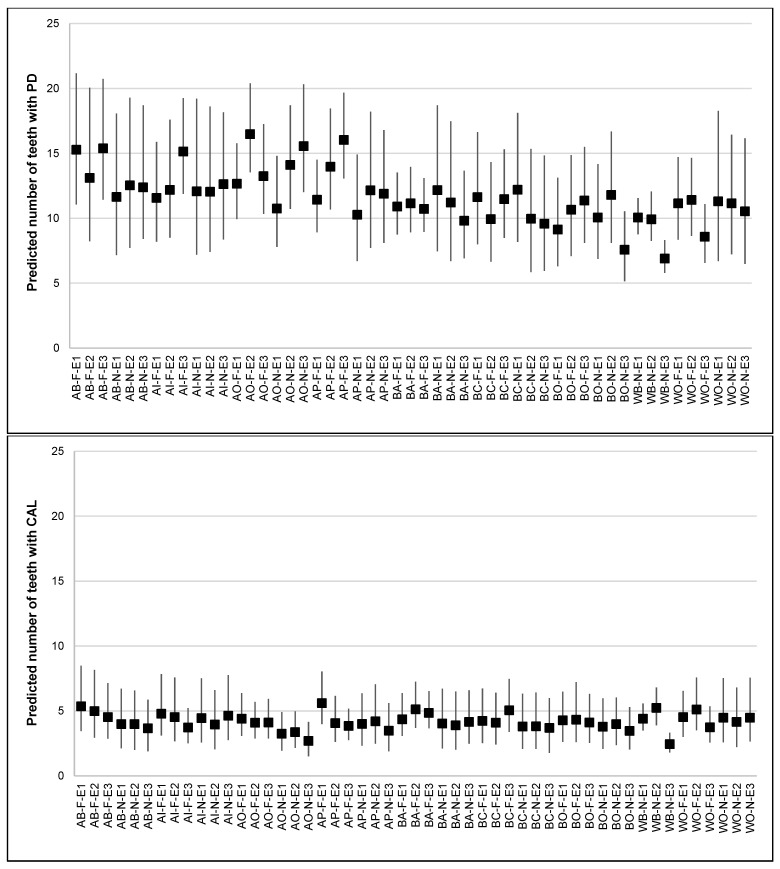
Predicted numbers of teeth with PD (**top plot**) and CAL (**bottom plot**), by intersectional strata defined by ethnicity (AB: Asian Bangladeshi, AI: Asian Indian, AO: Asian Other, AP: Asian Pakistani, BA: Black African, BC: Black Caribbean, BO: Black Other, WB: White British, and WO: White Other), nativity status (N: Native; F: Foreign) and education (E1: low, E2: medium, and E3: high). Predictions were based on the simple intersectional model. Markers indicate estimated effects, and whiskers indicate 95% credible intervals.

**Figure 2:**
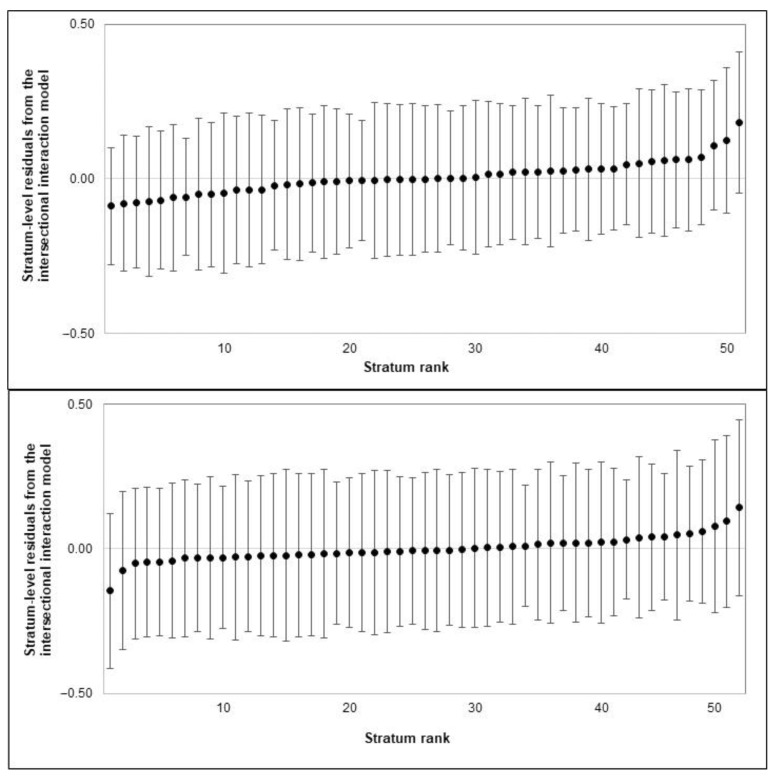
Intersectional interaction effects of ethnicity, nativity status and education on the numbers of teeth with PD (**top plot**) and CAL (**bottom plot**). Stratum residuals are the remaining difference between total predicted values for each stratum and stratum-level predictions based on additive effects only. Markers indicate estimated effects, and whiskers indicate 95% credible intervals. Intersectional strata were ordered according to their interaction effect. Negative and positive values indicate the number of teeth affected was below and above predicted scores for a given stratum, respectively.

The results from MAIHDA analysis are shown in Table 2. The VPC from the simple intersectional model indicated that 5% of the total variance in the number of teeth with PD was attributed to between-stratum differences. Figure 1 shows between-stratum differences, with the largest and smallest predicted numbers of teeth with PD seen among foreign-born Asian Other adults with medium education (16.5) and native-born White British adults with high education (6.9), respectively. The greatest numbers of teeth with PD were often found among foreign-born Asian adults with high education, whereas the smallest numbers were found among native-born White adults with high education. The known reverse gradients in periodontitis by education were only observed among White and native-born Black adults. The PCV from the intersectional interaction model indicated that 74% of the observed stratum-level variation was due to the additive effects of ethnicity, nativity status, education and confounders. Every Asian minority (Pakistani, Indian, Bangladeshi and Other) and foreign-born adults had more teeth with PD than did White British and native-born adults, respectively. However, no differences were found between education groups. The stratum-level residuals showed that all interaction effects were very small and not statistically different from zero (Figure 2).

Furthermore, the VPC from the simple intersectional model for the number of teeth with CAL indicated that 2.1% of the total variance resides at the intersectional strata level. The predicted numbers of teeth with CAL for every stratum are shown in Figure 2, with the largest and smallest predicted numbers found among foreign-born Pakistani adults with low education (5.6) and native-born White British with high education (2.4), respectively. The greatest number of teeth with CAL were found among ethnic minority, foreign-born adults with low education. Reverse gradients by education were observed in almost all ethnic groups. The PCV from the intersectional interaction model indicated that 74% of the between-stratum variance was due to the additive effects of ethnicity, nativity status, education and confounders. Foreign-born adults had more teeth with CAL than native-born adults did. In addition, Asian Other adults and those with high education had fewer teeth with CAL than White British adults and those with low education, respectively. The stratum-level residuals showed that all interaction effects were not statistically different from zero (Figure 2).

## 4. Discussion

This study found a complex pattern of social inequalities in periodontal status characterised by high heterogeneity between intersectional strata and outcome specificity. Intersectional strata differ markedly from each other with respect to their predicted number of teeth with PD, but much less so with respect to their predicted number of teeth with CAL. It was not the intersectional strata experiencing multiple dimensions of marginalisation but those experiencing a mix of disadvantage and privilege, such as foreign-born Asian adults with high education, who had the greatest burden of disease. This finding implies that the protective effects of education do not counterbalance the increased periodontitis risk associated with other social exposures (ethnicity and nativity status). Whilst we found evidence of substantial inequalities in periodontal measures between strata, only a modest share of the total variance (3–5%) was attributable to between-stratum differences. This finding suggests that these intersectional identities (strata membership) poorly predict variations in periodontal measures at the individual level and that other dimensions of social stratification could be more relevant but have yet to be identified as such.

We also found that differences between intersectional strata were largely (73–74%) driven by the additive (as opposed to the interaction) effects of ethnicity, nativity status and SEP. Although some intersectional strata had strata-level residuals above or below zero (implying their expected numbers of affected teeth were higher or lower than expected based solely on additive effects), the 95% credible intervals for all residuals crossed the zero value. This means that the magnitude and direction of the effects of ethnicity, nativity status and SEP were roughly similar across all intersectional social strata, thus rejecting the intersectionality hypothesis in relation to periodontal status. Although social determinants, such as racism, classism and xenophobia, engender interlocking systems of oppression and privilege that mould the day-to-day experiences and life opportunities of people because of their multidimensional social identities [17,18], their influence seems additive rather than multiplicative in our participants.

Of the three social identities evaluated, only nativity status was consistently associated with both periodontal measures. Immigrants face substantial structural issues when arriving to the UK, including discrimination and other major stressors in today’s political and social climate, all of which could affect their health [11]. However, the fact that the study sample was drawn from a largely deprived and ethnically diverse population could have played a role in these findings. Recruiting adults living in a deprived area restricted variability in SEP levels among participants and the ability to identify associations. Moreover, the residential segregation of ethnic minorities could benefit health by promoting social networks that provide emotional and instrumental support as well as shielding ethnic minorities from exposure to prejudice and discrimination, the so-called ethnic density effect [36]. Taken together, our findings suggest that the presence of intersectional interaction depends on both context and outcome, thus highlighting the value of confirming intersectional findings across multiple settings and various health outcomes.

From a public health perspective, our findings suggest that whole-population strategies might be more relevant than selective strategies for specific intersectional social strata. Universal policies and large-scale programmes may be beneficial to reduce health inequalities for many noncommunicable diseases, including periodontitis, and for all intersectional strata. Based on our experience, we believe an MAIHDA approach to explore intersectional social strata is a better theoretical and analytical framework for the evaluation of social inequalities in health compared to unidimensional analyses of gradients in health. Because substantial inequalities were present according to ethnicity, nativity status and SEP, these social dimensions should be considered in future research and public health practice.

There are some limitations to this study. First, we analysed cross-sectional data, which precludes drawing any causal inferences. Second, we did not use survey weights as they cannot be applied with MCMC estimation methods. Therefore, our findings cannot be extrapolated beyond the study sample. Third, the MAIHDA approach requires large and diverse samples. As MAIHDA estimates tend to be conservative for strata with few participants, a larger sample could yield strata-level residuals that are significant. Fourth, while ethnicity, nativity status and SEP are salient social identifies in relation to periodontitis, findings could differ if other social identities are considered. Two variables are worth mentioning here. We chose education as our SEP indicator because it reflects life chances and can determine both international migration and social standing. We also used country of birth as a crude indicator for immigration status. Whether other indicators of SEP and the legal status of immigrants (documented and undocumented) intersect with ethnicity and nativity status to engender health inequalities requires further exploration.

## 5. Conclusions

This study found little support for the intersectionality hypothesis in relation to periodontal status. Although substantial social inequalities were found between intersectional strata, most of the variation in periodontal measures was at the individual rather than stratum level, and a large proportion of the between-stratum variation was explained by the additive, not interaction, effects of ethnicity, nativity status and SEP.

## Figures and Tables

**Table 1:** Description of the study sample and periodontal measures by explanatory variables.

Explanatory Variables	Study Sample (*n* = 1936)		Teeth with PD		Teeth with CAL	
	*n*	%	Mean	(SD)	Mean	(SD)
Ethnicity						
White British	547	28.3%	8.7	(8.7)	6.2	(3.9)
White Other	158	8.2%	9.3	(8.7)	6.3	(4.9)
Black African	286	14.8%	10.5	(8.7)	7.6	(5.0)
Black Caribbean	90	4.7%	9.6	(8.2)	5.1	(4.1)
Black Other	137	7.1%	8.6	(8.0)	6.1	(3.5)
Pakistani	206	10.6%	14.2	(11.0)	7.6	(4.4)
Indian	112	5.8%	14.7	(10.5)	7.5	(4.5)
Bangladeshi	73	3.8%	17.4	(8.9)	7.6	(5.5)
Asian Other	327	16.9%	14.9	(11.1)	5.8	(3.3)
*p*-value ^a^			*<0.001*		*0.158*	
Nativity						
Native-born	873	45.1%	9.8	(9.4)	5.9	(3.4)
Foreign-born	1063	54.9%	12.7	(10.1)	7.1	(4.8)
*p*-value ^a^			*<0.001*		*<0.001*	
Education						
Low	652	33.7%	10.7	(9.2)	6.9	(4.6)
Medium	490	25.3%	12.2	(10.3)	7.5	(4.7)
High	794	41.0%	11.3	(10.2)	5.8	(3.4)
*p*-value ^a^			*0.154*		*0.003*	
Sex						
Men	623	32.2%	12.1	(10.0)	5.0	(7.1)
Women	1313	67.8%	11.0	(9.8)	3.8	(6.4)
*p*-value ^a^			*0.103*		*0.002*	
Age group						
16–24 years	165	8.5%	9.1	(9.4)	1.7	(4.5)
25–34 years	712	36.8%	12.0	(10.1)	3.6	(6.5)
35–44 years	710	36.7%	11.0	(10.1)	3.8	(6.2)
45–54 years	208	10.7%	11.8	(9.1)	6.6	(7.6)
55–65 years	141	7.3%	11.9	(9.1)	8.1	(7.7)
*p*-value ^a^			*0.375*		*<0.001*	

^a^: from a simple negative binomial regression model fitted for each periodontal measure.

**Table 2:** Results from the cross-classified multilevel analysis of individual heterogeneity and discriminatory accuracy (MAIHDA) for periodontal measures (*n* = 1936).

	Teeth with PD		Teeth with CAL	
	Model 1A ^a^	Model 1B ^a^	Model 2A	Model 2B
	RR (95% CI)	RR (95% CI)	RR (95% CI)	RR (95% CI)
Fixed effects (*n* = 1936 adults)				
Ethnicity (reference group: White British)				
White Other		1.07 (0.81–1.39)		0.96 (0.63–1.36)
Black African		1.19 (0.92–1.61)		1.12 (0.77–1.67)
Black Caribbean		1.11 (0.78–1.47)		0.80 (0.46–1.27)
Black Other		1.03 (0.78–1.35)		0.83 (0.53–1.20)
Pakistani		1.62 (1.22–2.14)		0.89 (0.61–1.25)
Indian		1.70 (1.25–2.30)		0.94 (0.60–1.49)
Bangladeshi		2.17 (1.51–3.04)		1.11 (0.65–1.79)
Asian Other		1.77 (1.40–2.26)		0.70 (0.50–0.94)
Nativity status (reference group: Native-born)				
Foreign-born		1.16 (1.01–1.30)		1.60 (1.24–1.92)
Education (reference group: Low)				
Medium		1.14 (0.95–1.36)		1.03 (0.81–1.35)
High		0.98 (0.84–1.17)		0.74 (0.60–0.91)
Sex (reference group: Men)				
Women		0.93 (0.84–1.02)		0.81 (0.66–0.96)
Age groups (reference group: 16–24 years)				
25–34 years		1.29 (1.07–1.46)		2.19 (1.73–2.78)
35–44 years		1.23 (1.02–1.41)		2.48 (1.97–3.06)
45–54 years		1.40 (1.12–1.69)		4.08 (2.91–5.46)
55–65 years		1.39 (1.08–1.75)		4.90 (3.41–6.57)
Intercept	11.39 (10.41–12.47)	6.47 (5.27–8.02)	4.01 (3.43–4.62)	1.76 (1.29–2.29)
Random effects (*n* = 51 intersectional social strata):				
Between-stratum variance	0.06 (0.03–0.12)	0.02 (0.003–0.04)	0.08 (0.02–0.19)	0.02 (0.001–0.07)
VPC (%)	4.8%	1.3%	2.1%	0.6%
PCV (%)		73.6%		74.0%

CAL: clinical attachment loss; PD: probing depth; PCV: percentual change in variance; RR: rate ratio; VPC: variance partition component. ^a^ Two-level negative binomial regression models were fitted for each periodontal measure, with participants clustered within intersectional social strata. Model A is the simple intersectional model (variance components model), and Model B is the intersectional interaction model (random intercepts model).

## Data Availability

Data could be made available upon a reasonable request to the corresponding author.
